# Towards the Integration of an Anti-Contractile Compound Within Drug-Coated Balloon Therapy

**DOI:** 10.1007/s13239-025-00798-7

**Published:** 2025-07-25

**Authors:** Dima BaniHani, John F. Eberth, Francis G. Spinale, Vipul C. Chitalia, Jahid Ferdous, Vijaya B. Kolachalama, Tarek Shazly

**Affiliations:** 1 Department of Biomedical Engineering, College of Engineering and Computing, University of South Carolina, Columbia, SC 29208 USA; 2 Biomedical Engineering, Science and Health Systems, Drexel University, Philadelphia, PA 19104 USA; 3 Columbia VA Health Care Center, VA, Columbia, SC 29208 USA; 4 Department of Cell Biology and Anatomy, School of Medicine, University of South Carolina, Columbia, SC 29208 USA; 5 Center of Cross-Organ Vascular Pathology, Department of Medicine, Boston University School of Medicine, Boston, MA 02118 USA; 6 VA Boston Healthcare System, Boston, MA 02115 USA; 7 Department of Medicine, Boston University Chobanian & Avedisian School of Medicine, Boston, MA 02118 USA; 8 Department of Biomedical Engineering, Bangladesh University of Engineering and Technology, Dhaka, 1205 Bangladesh; 9 Department of Computer Science and Faculty of Computing & Data Sciences, Boston University, Boston, MA 02115 USA; 10 Department of Mechanical Engineering, College of Engineering and Computing, University of South Carolina, Columbia, SC 29208 USA

**Keywords:** Drug-coated balloon, Paclitaxel, Valsartan, Anti-contractile, Urea

## Abstract

**Purpose:**

Drug-coated balloon (DCB) therapy is a promising approach to treat peripheral artery disease (PAD), wherein lesion site preparation, balloon inflation, and the local delivery of anti-proliferative drugs such as paclitaxel (PTX) restores and retains lumen patency. Although largely successful in PAD applications, broader clinical deployment is in part limited by the occurrence of late lumen loss due to inward vessel remodeling at the treatment site, a maladaptive chronic response that has been clinically-observed to coincide with elevations in resident vascular smooth muscle cell (vSMC) tone. This study aims to explore a novel strategy to improve DCB efficacy via drug-based attenuation of vSMC tone at the treatment site.

**Methods:**

As a strategy to mitigate this post-DCB failure mode, we consider the local co-delivery of PTX and an additional drug that induces relaxation of vSMCs, specifically the clinically-approved anti-hypertensive drug valsartan (VAL). The potential benefit of drug-based regulation of vSMC tone is supported by recent theoretical studies that predict inward remodeling in the presence of hypertension and endothelial cell dysfunction, both common co-morbidities in PAD patients and established causes of elevated vSMC contractility. The specific selection of VAL as the anti-contractile payload constituent is motivated by its well-known pharmacokinetic and safety profiles, and the notion that current clinical use and familiarity could promote rapid translation in the context of DCBs.

**Results:**

Our obtained results quantify the potency of VAL to induce local vSMC relaxation in arterial tissue, demonstrate the feasibility of PTX and VAL co-delivery using the canonical excipient urea for balloon coating formation, and elucidate key structure-function relations to facilitate efficient drug delivery with these novel coatings.

**Conclusion:**

Our study supports the continued evaluation of VAL for inclusion in DCB formulations due to its potential to redirect post-treatment arterial remodeling. Future in-vivo studies which examine the co-delivery of PTX and VAL in the context of DCBs are needed to establish both the safety and efficacy of this novel approach.

## Introduction

Several limitations of permanent drug-eluting stents (DES) [1, 2] are inherently mitigated by the leave-nothing-behind strategy embodied in drug coated balloon (DCB) therapy, in which lesion site preparation followed by transient (~ 2–3 min) intravascular balloon inflation both addresses the offending lesion and locally delivers an anti-proliferative drug, most commonly paclitaxel (PTX), to the arterial wall [3–8]. Thus, with no permanent implant to confound post-procedural arterial healing, DCB therapy offers a means to acutely restore (via initial lesion preparation and balloon-mediated lesion compression) and subsequently maintain (via delivered PTX activity) vessel patency. DCBs have seen extensive use in the treatment of peripheral artery disease (PAD), which affects over 8 million Americans, and as such represent a device class of high clinical significance [9–12]. However, their comparatively sparse use beyond the peripheral circulation and the incomplete understanding of the key determinants of therapeutic efficacy [13–16] indicate that DCB clinical potential has not been fully realized.

Clinical hesitation for broader DCB use, specifically within the coronary circulation, can be partly attributed to inconsistent and unpredictable long-term treatment outcomes, including the occurrence of late lumen loss due to inward arterial remodeling at the deployment site [17]. Based on experimental observations of maladaptive inward arterial remodeling, including positive associations among vSMC tone, endothelial cell (EC) dysfunction, and the degree of inward wall growth [18–21], we rationalize that drug-based induction of local vSMC relaxation can shift the direction of remodeling-mediated growth from inward-to-outward and insomuch prevent subsequent loss of lumen patency. Although experimentally/clinically supported by only phenomenological relations, this notion aligns with our previous theoretical studies that predict the influence of vSMC tone on pressure-mediated arterial remodeling in the presence of EC dysfunction [22], where the ratio between vSMC synthetic output and contractile force generation is a deterministic factor of remodeling outcomes, including the wall growth direction. Hypertension and EC dysfunction, the latter of which may be exacerbated during lesion preparation [22, 23], are common PAD co-morbidities that could vary on a patient-specific basis and thus partially underlie the inconsistent outcomes limiting DCB therapy.

All approved DCBs contain anti-proliferative drugs, primarily PTX, sirolimus, or associated derivatives, as the bioactive payload component. We propose to augment the traditional payload with compounds that relax resident vSMCs during the early stages of post-DCB remodeling. Our approach builds upon previous studies that have explored the concept of modulating vSMC tone with nitric oxide donor molecules [24–26] in the context of endovascular interventions, including their local delivery via DCBs [24] and nanoparticle-based systems [26]. Moreover, DES-based delivery of angiotensin receptor blockers (ARBs) has been explored in clinical trials, with results that support the potential for remediation of inward growth [27–29].

In this study we examine novel coatings for co-delivery of PTX and the ARB drug valsartan (VAL). VAL (brand name Diovan) is clinically approved, well-characterized, and commonly prescribed as an anti-hypertensive, oral medication [30, 31]. VAL inhibits angiotensin II (AngII), increases the release of the vasodilating molecule bradykinin, and leads to a reduction in patient blood pressure [30]. Ang-II receptor blocking also prevents activation of pro-inflammatory pathways such as NF-κB, lowers oxidative stress through a reduction in reactive oxygen species, improves endothelial function by increasing NO bioavailability, and reduces immune cell recruitment and levels of pro-inflammatory cytokines such as IL-6 and TNF-α. Additionally, VAL does not impact norepinephrine activity and has known profiles in toxicity, binding, and functional antagonism [32–35]. Finally, due to antagonism of AngII receptors, VAL provides anti-proliferative effects [36, 37] that may work in concert with PTX to prevent post-DCB maladaptive remodeling, making it a particularly attractive compound to repurpose for DCB therapy.

In this study, we examined the feasibility of PTX and VAL co-delivery using urea-based coatings amenable to DCB applications. We first quantified the potency of VAL to induce vSMC relaxation via isometric analyses of 1-D active/passive mechanical testing data obtained on ring samples of porcine femoral artery. We then synthesized a series of urea-based coatings for PTX + VAL delivery, with titrated drug formulations featuring increasing VAL content. Using these coatings, we simulated DCB deployment with 1-D coating-tissue contact studies and measured the drug transfer efficiencies of both PTX and VAL to the arterial wall. Finally, we quantified microstructural (via scanning electron microscopy/image analysis of formed coating) and mechanical (via the recorded and modeled viscoelastic response of coating-tissue interfaces over the simulated DCB deployment period) differences among urea + PTX + VAL coatings and identified key structure-function relations, thus informing future coating design. By incorporating VAL, we aim to create payloads that mitigate inward arterial remodeling and thus preserve lumen patency following DCB treatment, providing a foundation for continued device development and ultimately improved clinical performance.

## Methods

### VAL-Induced Relaxation of vSMCs

All tissue sourcing used in this study is from animals designated for human consumption and thus not subject to IACUC protocols. The femoral artery (FA) was carefully isolated from a freshly slaughtered adult American Yorkshire sow (~ 3 year old, ~ 200 kg) and immediately transported to the laboratory in a phosphate buffered saline (PBS, LC Laboratories) solution. Upon arrival, the FA was further rinsed with PBS, cleaned of fat and connective tissue under a dissecting microscope, and cut transversally into cylindrical rings. Five rings (~ 4 mm outer diameter, ~ 0.5 mm wall thickness, and ~ 2 mm wall length) were prepared and placed into PBS until testing.

To initiate testing, a ring sample was mounted on horizontally-oriented 25-gauge cannulas controlled by the upper and lower arms of a uniaxial mechanical tester (Bose Electro Force 5270) [38]. The traction-free geometry was then measured with digital calipers to provide a basis for subsequent calculation of tissue stretch at each deformed state. The mounted sample was mechanically preconditioned with three tensile displacement cycles (displacement rate of 0.01 mm/s; stretch of 1.0–1.4) to achieve an elastic response. An identical fourth cycle was then performed, during which the integrated system software (Bose, Wintest) recorded the sample load and displacement data (100 data points/s). These data were subsequently processed to yield circumferential stress ($\:{\sigma\:}_{\theta\:}$) and circumferential stretch ($\:{\lambda\:}_{\theta\:}$) data reflective of the basal mechanical response of the tissue [38, 39].

To induce vSMC maximal contraction, VAL-mediated relaxation, and complete relaxation, stock solutions of epinephrine (EPI, LC Laboratories), VAL (LC Laboratories), and sodium nitroprusside (SNP, LC Laboratories), respectively, were prepared via dissolution in 1% dimethylsulfoxide (DMSO, LC Laboratories). Rings were submerged in 10^− 5^ M EPI for 15 min (to allow acclimation and manifestation of maximal vSMC contractility) and then mechanically preconditioned/tested as described above. Next, rings were rinsed with PBS, and then resubmerged, acclimated, preconditioned, and tested with solutions of increasing VAL concentration created via 5-fold serial dilutions to the stock solution (1 nM, 6 nM, 33 nM, 168 nM, 840 nM, 21 µM). To assess the passive mechanical response of fully relaxed vSMCs, the procedure was repeated using a 10^− 5^ M SNP solution [40–44].

### Preparation of Urea-Based PTX + VAL Coatings

Solutions for coating formation were prepared with the excipient urea (Sigma-Aldrich) and the drugs PTX and VAL in accordance with established protocols [45–48]. Urea was dissolved in ethanol (200 proof) at an agitation rate of 200 rpm for 6 h (1000 mg/ml solution concentration). Drug components were also dissolved in ethanol (200 proof) to yield a PTX solution (35 mg/mL) and a series of VAL solutions (8.75, 17.5, and 35 mg/ml). PTX + VAL solutions were then prepared by 1:1 v/v mixing of the PTX solution with each of the VAL solutions, thus yielding co-drug solutions with constant PTX content and titrated VAL content. Finally, these drug solutions (along with a PTX-only solution) were mixed with the urea solution (1:1 v/v) to provide excipient + drug solutions for subsequent spray coating. These urea-based coating variants are hereafter distinguished by the dosing ratio of VAL to PTX, namely [VAL/PTX] = 0, 0.25, 0.5, and 1.

To provide a ballon surface surrogate, nylon-12 films (0.5 mm thick) were punched into 8 mm circular disks, sonicated twice in a water-soap wash at room temperature to remove any potential surface particles, and then dried/stored at room temperature under controlled humidity. A spray-coater was used to deposit the prepared coating variant solutions onto the disk surfaces, with spraying distance (12.5 cm) selected to uniformly cover the disk surface area (50.2 mm^2^). The formed coatings were dried and kept in a desiccator at 25 °C for 48 h before use. The four resultant urea-based coatings consist of a PTX surface load of 3 ug/mm^2^ (all coatings) and a VAL surface load of 0, 0.25,1.5, or 3 ug/mm^2^.

### Coating Surface Microstructure

Scanning electron microscopy (SEM) and quantitative image analyses on the coating variants were used to assess composition-microstructure relations. SEM was preceded by gold-palladium nanoparticles sputter-coating (10 kV, 20 mA) onto the samples, resulting in an ~ 75 nm conductive coating that enabled high-magnification imaging (700× and 1200×) and visualization of surface microstructural features.

Microstructural coating features were quantified with a commercial image analyses software (ImageJ) applied to SEM images (32-bit grayscale), with uniformly applied image preprocessing steps that included: application of a median filter to remove edges, randomized image cropping to isolate 250 × 250-pixel regions-of-interest (ROI), and R-value intensity scoring across an ROI. Computed surface metrics utilize software-generated ROI gradient analyses, and included arithmetical mean deviation (Ra), root mean square deviation (Rq), kurtosis (Rku), and skewness (Rsk) of the R-value intensity distribution [49–51]. All surface metrics were normalized by value obtained for the [VAL/PTX] = 0 coating variant and reported as relative values.

### Acute Drug Transfer to Arterial Tissue

We configured a uniaxial mechanical testing apparatus (Bose Electro Force 5270) and programmed associated system software (Wintest) to simulate coating-tissue contact during DCB deployment. As previously described [47–48], a uniaxial interfacial compression test between coating variants and excised FA biopsies was initiated with establishment of a no-force (< 0.01 N) surface-to-surface contact, after which an interfacial compression (initial compression force of 3 ± 0.1 N) was applied for 180 s using a controlled ramped displacement (0.1 mm/s) and a positional dwell, with continuous capture (50 data points/sec) of interfacial load data. After completion of the programmed contact, the two test sample components (coating and tissue) were separated under a constant displacement rate (0.1 mm/s), unmounted, and prepared for the high-performance liquid chromatography (HPLC) to measure acute drug transfer.

Test sample components (coating and tissue) were immersed in HPLC-grade methanol and 0.1% (v/v) acidic acid, and then vortexed for 1 min. Acid was added to prevent drug transesterification in methanol. The samples were then sonicated twice for 30 min in a water bath sonicator, vortexed for 3 min between each run, and centrifuged at 5000 rpm for 10 min. The supernatant containing the extracted drugs was transferred to a new experimental tube and diluted in methanol. The extraction protocol was validated for each substrate (coated balloon surfaces and arterial vessel samples) and drug type (PTX and VAL) with controls containing known amounts of PTX and VAL.

### Tissue-Coating Contact Mechanics

The (constant) displacement - (variable) load data captured during tissue-coating contact studies (Sect. Acute Drug Transfer to Arterial Tissue) indicated significant stress relaxation, motivating the use of a standard linear solid (SLS) model of viscoelastic solids to describe interfacial contact mechanics [52]. Obtained data were transformed to depict the stress relaxation response of the coating-tissue test elements and modeled in accordance with SLS elements, namely an equilibrium spring with stiffness $\:{K}_{e}$ [kPa], a Maxwell spring with stiffness $\:{K}_{1}$ [kPa], a Maxwell dashpot with viscosity $\:\eta\:$ [kPa-sec], and a relaxation time constant $\:\tau\:$ [sec], where $\:\tau\:=\frac{\eta\:}{{K}_{1}}$. Nonlinear least-squares regression was performed to identify parameters SLS model predictions for test elements containing each coating variant (*N* = 5/variant), enabling quantification of the degree and kinetics of viscoelastic behavior manifested over the simulated deployment period. The SLS-based governing constitutive equation of stress relaxation, i.e. $\:\sigma\:\left(t\right),\:$ is

1 $$\sigma \left( t \right)~={\varepsilon _0}\left[ {{K_e}+{K_1}{e^{\frac{{ - t}}{\tau }}}} \right]$$

where $\:{\epsilon\:}_{0}$ is the (constant) compressive strain of the tissue-coating interface during the programmed positional dwell period.

### Statistical Analysis

All descriptive data are presented as the mean and standard error of the mean of measurements obtained across samples (*N* = 5 per experimental/control group). An analysis of variance (ANOVA) and unpaired t-test were used to examine the statistical significance of the obtained data. A Pearson’s correlation coefficient ($\:\rho\:$) was used to quantify the degree of correlation between experimental response variables. A statistically significant difference/correlation was defined when p-value < 0.1.

## Results

### VAL-Based Alteration of Arterial Mechanical Response

**Fig. 1:**
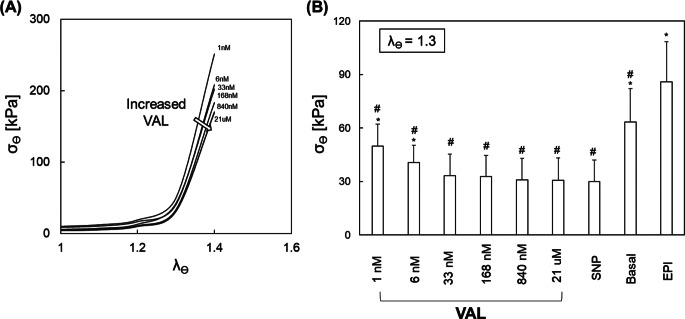
VAL-mediated vSMC relaxation. (**A**) Representative circumferential stress-circumferential strain response exhibited by a porcine femoral artery (FA) ring sample under increasing VAL concentration, wherein the observed rightward shift in the response curves indicates that VAL diminishes active stress generation. (**B**) Isometric comparison of the circumferential stress response of porcine FA ring samples under increasing VAL concentration, as well as in the fully-relaxed (SNP), basal, and maximally-contracted (EPI) vSMC states. *indicates *p* < 0.05 vs. SNP, ^#^indicates *P* < 0.05 vs. EPI. *N* = 5/group; error bars represent + SEM

The circumferential stress-stretch response of arterial ring samples under increased VAL dosing (Fig. 1A) retained canonical soft tissue nonlinear behavior, with a dose-dependent rightward shifting of the response curves. The samples were stretched to a stretch ratio of 1.3, resulting in a 30% increase in length. In (Fig. 1B) the average circumferential stress decreased monotonically as VAL concentrations increased. The fully relaxed (SNP) and maximally contracted (EPI) vSMC states demonstrate that a relatively low VAL concentration (168–840 nM) completely attenuates vSMC contractile force generation in an acute setting.

### Coating Morphology

**Fig. 2:**
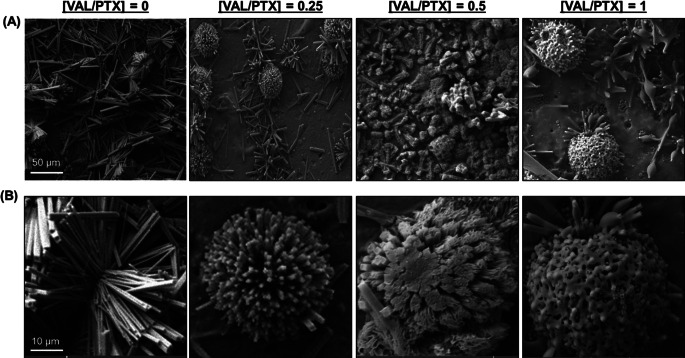
Urea-based coating surfaces visualized via scanning electron microscopy (SEM). (**A**) Low- and (**B**) high-magnification SEM images obtained for urea-based coatings with titrated VAL content. Qualitative evaluation of urea-based coatings confirms the presence of needle-like features on urea-PTX (left panels) and suggests that upon incorporated VAL aggregates on and progressively dulls these characteristic features

**Fig. 3:**
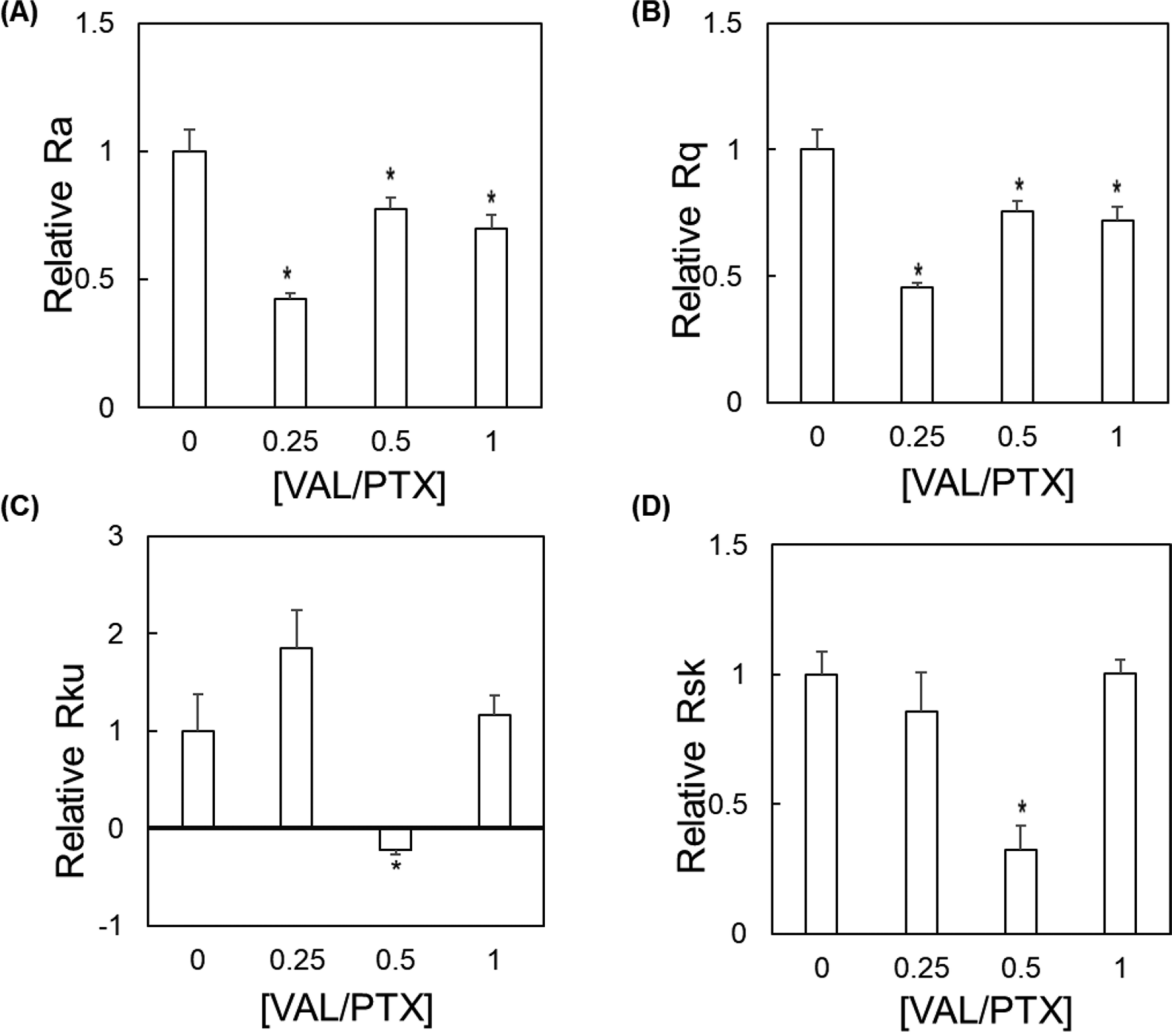
Urea-based coating microstructure. Quantitative SEM image analysis based on a statistical description of R-value intensity scoring within a randomly selected region-of-interest was performed to generate relative metrics describing the coating microstructure, including the (**A**) arithmetical mean deviation (Ra), (**B**) root mean square deviation (Rq), (**C**) kurtosis (Rku), and (**D**) skewness (Rsk). * indicates *p* < 0.05 vs. [VAL/PTX] = 0. *N* = 5/group; error bars represent + SEM

SEM assessment of coating surface morphology qualitatively showed that the previously reported [48] and herein confirmed needle-like microstructure of urea-PTX coatings is evidently dulled with incorporation of VAL, which appears to progressively coalesce on the needle edges/ends as drug content is increased (Fig. 2). These microstructural changes were quantified via SEM image analysis, where changes in R_a_ and R_q_ show that VAL-mediated feature dulling is associated with significant reductions in the mean and dispersion of feature heights over the ROI (Fig. 3A-B). Higher-order descriptors of surface morphology, including R_ku_ and R_sk_, are significantly altered at intermediate VAL dosing (Fig. 3B-C, [VAL/PTX] = 0.5]).

### Acute Drug Transfer

**Fig. 4:**
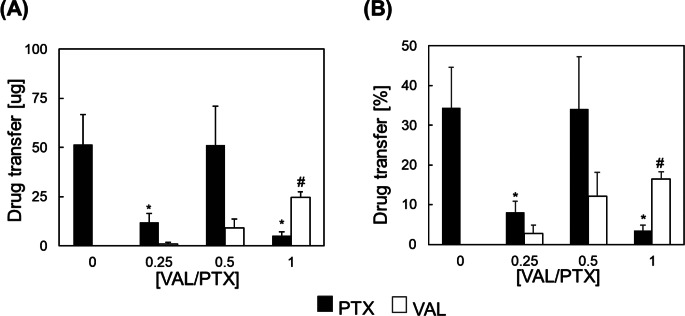
Acute transfer of urea-based coating drug components (PTX and VAL) to arterial tissue. (**A**) Total drug mass and (**B**) percentage of initial loading transferred to the arterial tissue in an ex-vivo simulation of DCB deployment. * indicates *p* < 0.05 vs. [VAL/PTX] = 0. # indicates *p* < 0.05 vs. [VAL/PTX] = 0.25. *N* = 5/group; error bars represent + SEM

The acute drug transfer from urea-based coatings to arterial tissue was assessed ex-vivo and reported as both the total mass of drug transferred to/retained within the arterial wall (Fig. 4A) and the percent transferred with respect to the initial coating composition (Fig. 4B), thus providing insight into local drug dosing potential as well as coating delivery efficiency, respectively. In the urea-PTX coating, greater than 30% of the payload (~ 50 µg) was transferred to the tissue during the deployment period. The amount and efficiency of PTX delivery were generally diminished with incorporation of VAL, with exception of the [VAL/PTX] = 0.5 variant in which both PTX transfer metrics were essentially unchanged while the amount and efficiency of VAL transfer increased with increasing [VAL/PTX] ratio.

### Coating-Tissue Intradeployment Mechanics

**Fig. 5:**
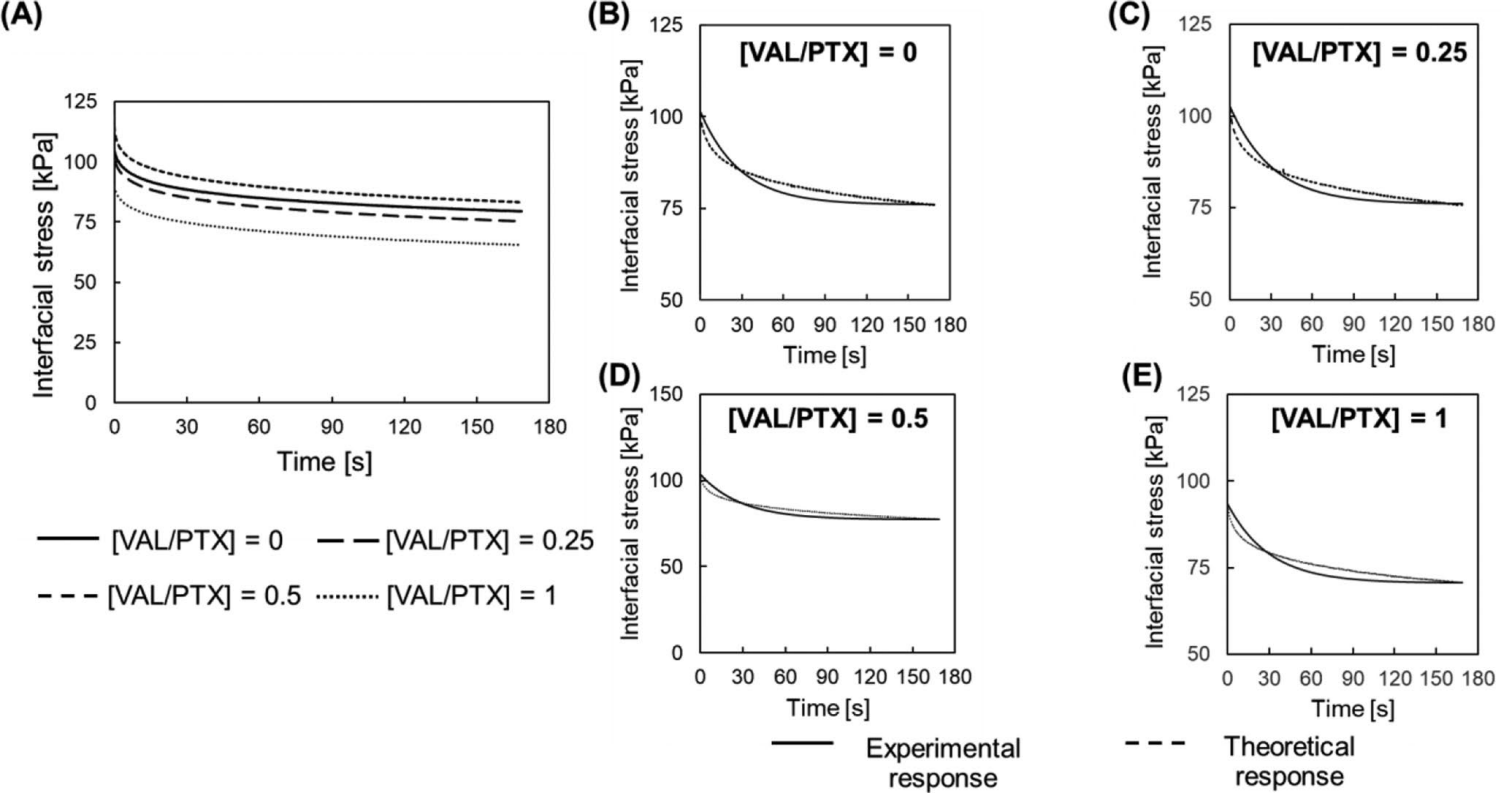
Stress relaxation response of the coating-tissue interface. (**A**) Mean stress relaxation response of coating-tissue interfaces. (**B-D**) Representative experimental vs. theoretical stress relaxation responses of coating-tissue interfaces exhibit excellent correlation for all coating variants (*R* > 0.97), supporting the use of an SLS to model interfacial mechanics

All coating-tissue constructs exhibited notable viscoelastic behavior over the simulated DCB deployment, with ~ 25% stress relaxation from the instantaneous value to equilibrium stresses of ~ 70–90 kPa (Fig. 5A; Table 1). The response data were amenable to modeling within the SLS framework, as evidenced by excellent agreement between theoretical and experimental stress relaxation curves (Fig. 5B-E). The single model time-constant $\:\tau\:$ was ~ 30 s for all coating variants, which is within the expected duration of DCB deployment.

**Table 1:**
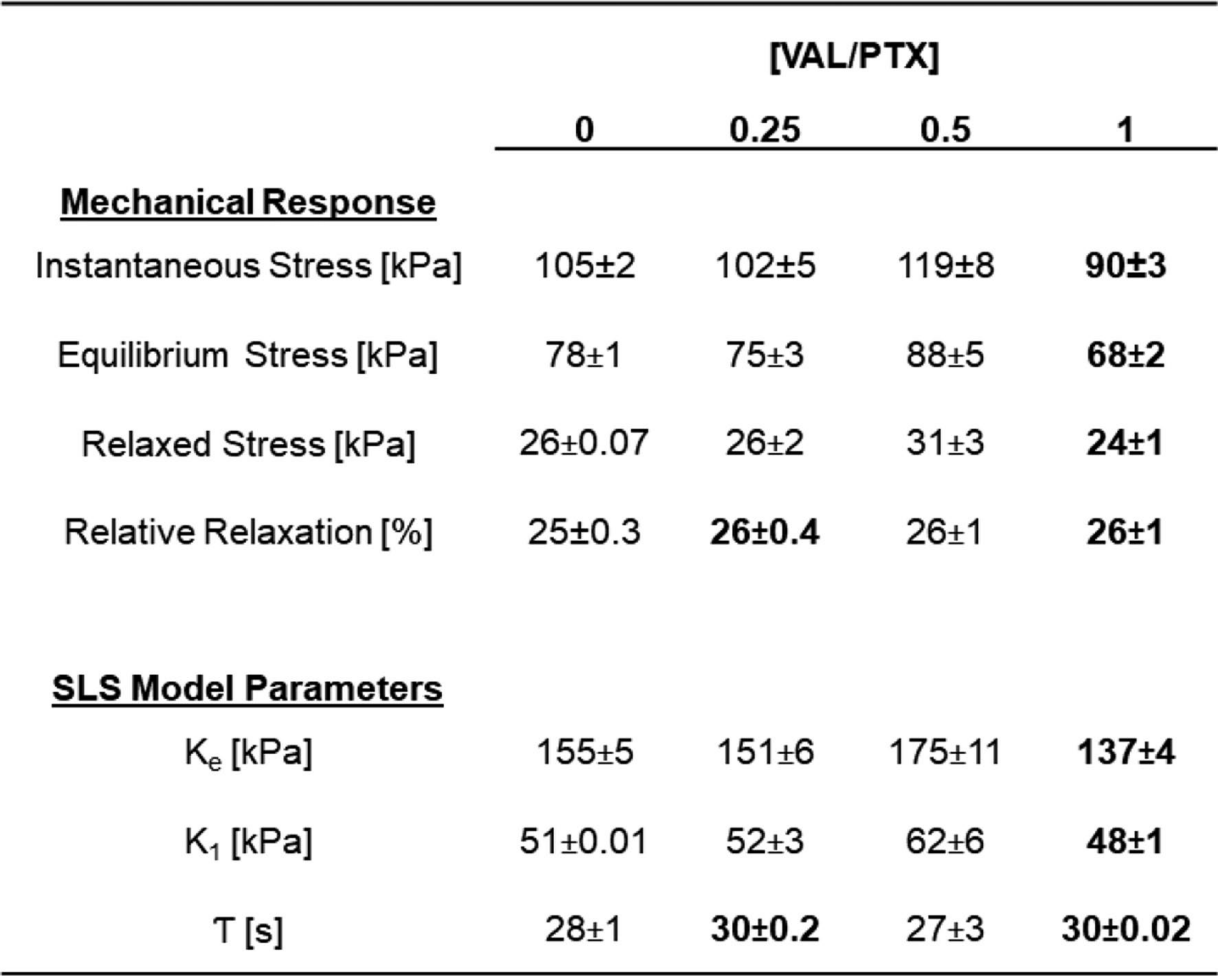
Coating-tissue interfacial mechanical response and associated SLS model parameters. All values reported as mean ± sem (*N* = 5). Bold font indicates *p* < 0.05 vs. [VAL/PTX] = 0

### Response Variable Correlation Analysis

**Fig. 6:**
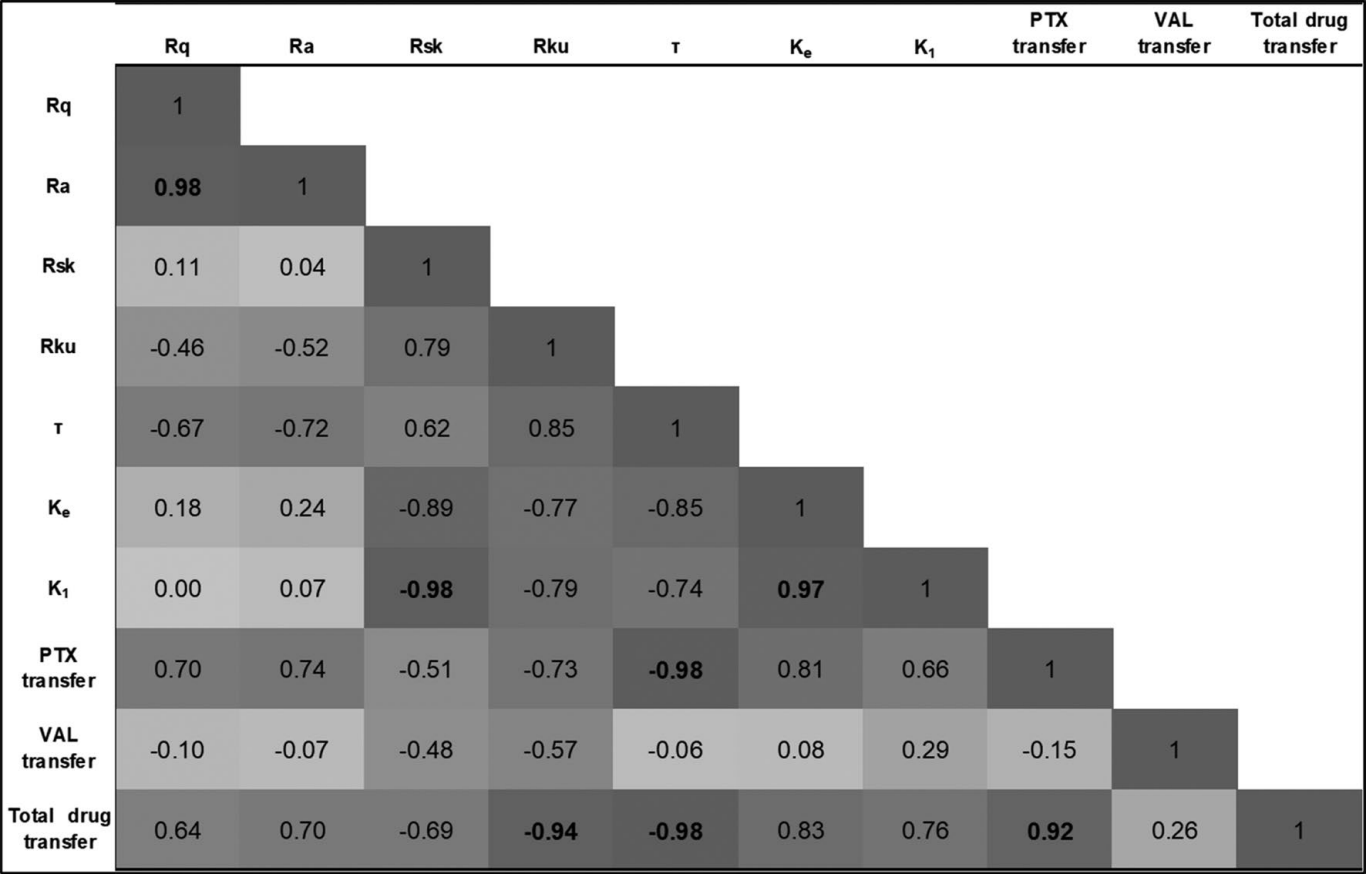
Pearson’s correlation coefficients between obtained experimental response variables. Bold font indicates statistically significant (*p* < 0.10) correlation between response variables

Correlations among experimental response variables are illustrated in (Fig. 6). Relatively strong (ρ > 0.7) positive correlations between PTX transfer and surface roughness (R_q_ and R_a_). Conversely, VAL exhibited a weak inverse correlation to surface roughness metrics, (Fig. 2). Negative correlations between R_sk_/R_ku_ and drug transfer efficiency (both PTX and VAL). The most significant determinant of PTX transfer was the SLS model time constant Ƭ, wherein strong inverse correlation (ρ = -0.98) between these variables suggests that increased interfacial mobility during the deployment period increases the effective diffusivity of the drug across the coating-tissue interface.

In terms of intradeployment mechanics, our findings suggest that increased interfacial stiffness (both instantaneous and equilibrium) promotes PTX transfer, while VAL transfer exhibits minimal correlation. These differential effects of interfacial stiffness on the PTX and VAL transfer may be due to how these drugs are incorporated into the coating microstructure, with the positive effects on PTX transfer potentially due to its comparatively uniform incorporation into the needle-like features and the expectation that less deformable coatings will promote feature penetration into the arterial wall during DCB deployment. Although not examined in these studies, this mechanical effect would also likely increase the initial depth of PTX delivery into the arterial wall and ultimately mitigate post-procedural drug washout from the application site.

## Discussion

Our study sought to establish a basis for integrating the anti-contractile drug VAL within a traditional DCB payload consisting of the anti-proliferative drug PTX and the excipient urea. We first assessed the dose-dependent VAL potency for acute induction of vSMC relaxation in ex- vivo mechanical studies (Sect. VAL-Induced Relaxation of vSMCs), which reflected drug-mediated vSMC relaxation in isometric comparisons (ʎ_ϴ_= 1.3) of the VAL dosing effects on the tissue stress response in the basal, fully relaxed (SNP), and maximally contracted (EPI) vSMC states, thus supporting an intended bioactive effect of this drug in DCB therapy. However, due to in-situ drug transport away from the treatment site and drug metabolism as expected in DCB applications, the observed acute efficacy of VAL may not persist for adequate time periods to effectively attenuate arterial remodeling, which will likely require higher initial tissue dosing. Nevertheless, these data support the proposed repurposing of VAL as a local vSMC relaxant, provide initial information to guide dosing strategies in the context of DCBs, and motivate the integration of this drug within the therapeutic payload.

The experimental design and synthesis of urea-based PTX + VAL coatings with titrated VAL content (Sect. Preparation of Urea-Based PTX + VAL Coatings) investigated how composition influences coating surface microstructure (Sect. Coating Surface Microstructure). All groups exhibited descriptors of surface roughness values significantly lower than those of the control group. Interestingly, the most significant reductions in these metrics were observed in the low VAL variant ([VAL/PTX] = 0.25), while higher VAL concentrations promoted additional coating formation and partial feature recovery by virtue of greater overall surface coverage. The negative kurtosis and diminished skewness of the [VAL/PTX] = 0.5 variant together indicate that the formed coating is comparatively uniform throughout the ROI and suggests it would confer enhanced coating-tissue contact in DCB applications.

The assessment of acute co-drug delivery efficiency to the arterial wall (Sect. Acute Drug Transfer to Arterial Tissue) provides information on coating delivery efficiency and local drug dosing potential, as well as confirming the relative efficiency conferred by the excipient urea when compared to other candidate materials in current DCBs [53, 54]. The amount and efficiency of PTX and VAL delivery with the former expected and the latter suggestive that the observed VAL accumulation on the needle-like coating features increases the contact area between it and the arterial wall during simulated deployment, and in doing so promotes diffusion-mediated drug transfer. Among the tested coating variants, [VAL/PTX] = 0.5 results in the greatest total drug transfer to arterial tissue and therefore shows most potential as a novel payload formulation. Future studies will explore if retention of this drug component ratio at higher dosing levels preserves favorable transfer kinetics, and if so could it provide a meaningful single variable (drug component loading) for tuning of the urea-based coatings that effectively control remodeling outcomes.

The viscoelastic behavior of the coating-tissue contact mechanics study (Sect. Tissue-Coating Contact Mechanics) revealed both the time course and extent of construct stress relaxation are in qualitative agreement with previously obtained data at similar (constant) strain levels for urea-based coatings [48]. The single SLS model parameters support the notion that interfacial mobility can potentially impact drug transfer kinetics.

We identify correlations among experimental response variables obtained in these studies to elucidate key property-function relations that can guide future DCB design (Sect. Statistical Analysis) and may provide insight into key phenomenological relations that can be used. Positive correlations between PTX transfer and surface roughness (R_q_ and Ra) suggest that diffusion is the dominant PTX transfer modality and thus central to traditional DCB delivery. Negative correlations between R_sk_/R_ku_ and drug transfer efficiency (both PTX and VAL) suggest that coating continuity promotes tissue contact and as a result enhances diffusion-mediated drug transfer. Taken together, these findings support the formation of rough and continuous surfaces to enhance essential coating function (drug transfer) in novel DCB applications.

### Study Limitations

While our results support the potential of VAL as a novel payload component to increase DCB efficacy, key study limitations should be considered in the interpretation of our results. Most importantly, these initial acute studies do not establish the post-procedural time over which VAL will remain at the deployment site and/or induce vSMC relaxation, nor that this intended alteration of tone will effectively redirect remodeling-mediated arterial wall growth from inward to outward. Although this concept is supported by previous theoretical studies, in-vivo evaluation of our novel DCBs will be required to establish functional gain and justify co-drug delivery strategies. Specifically, in-vivo studies in which inward remodeling is partially/fully attenuated by our DCBs despite complete local denudation would provide strong support for our proposed coating design strategy. Secondly, we evaluated only four coating variants (three with a VAL component), all of which had equivalent PTX content. The potential design space for PTX + VAL coatings is much larger than what was probed in our study, and it is possible that different structure-function relations (correlations) would emerge at higher overall dosing levels. Finally, our ex-vivo simulation of DCB deployment consists of uniaxial loading of the coating-tissue interface, which is not representative of the three-dimensional mechanics governing the actual procedure. The forces transmitted to the arterial wall during balloon angioplasty are primarily derived from the inflation pressure, which would induce radial compression in the arterial wall but also significant circumferential tension – this bi-axial deformation may impact local drug transport and is not accounted for in our experimental design. In DCB applications, additional mechanical factors, including the coating deformation/fracture during the balloon inflation, would impact coating-tissue contact mechanics and potentially modulate the influence of interfacial stress on drug delivery efficiency.

## Conclusion

This study explores a novel approach to improving the efficacy of DCB therapy, with the goal of repurposing the anti-hypertensive drug VAL as a payload component that relaxes resident vSMCs and ultimately prevents deleterious inward arterial remodeling. The key findings of the study are: VAL effectively induces vSMC relaxation in porcine FA, providing support for the intended function of this DCB component; PTX and VAL can be efficiently co-delivered using urea-based coatings, with the greatest delivery efficiency observed with equivalent initial drug contents; key microstructural and mechanical properties of urea + PTX + VAL coatings influence drug delivery efficiency, including descriptors of surface roughness/continuity and viscoelastic relaxation of the coating-tissue interface formed during DCB deployment. Future in-vivo studies which examine the co-delivery of PTX and VAL in the context of DCBs are needed to establish both the safety and efficacy of this novel approach to mitigate post-procedural inward arterial remodeling.
